# Supplementary material for: Iodine Status, Thyroid Function, and Birthweight: A Complex Relationship in High-Risk Pregnancies
Source: J Clin Med. 2020 Jan 8;9(1):177. doi: 10.3390/jcm9010177 (PMC7019691; doi:10.3390/jcm9010177)
Supplement: Supplementary file 1 [file jcm-09-00177-s001.pdf]

Supplementary Table 1: Correlations between Apgar and pH values in Low-risk and High-risk pregnancies.

**Low-Risk Pregnancies**	1-minute Apgar	5-minute Apgar	Arterial pH umbilical cord
5-minute Apgar	$r = 0.739$, $p < 0.001$		
Arterial pH umbilical cord	$r = 0.526$, $p < 0.001$	$r = 0.431$, $p < 0.001$	
Venous pH umbilical cord	$r = 0.417$, $p < 0.001$	$r = 0.311$, $p < 0.001$	$r = 0.793$, $p < 0.001$
**High-Risk Pregnancies**	1-minute Apgar	5-minute Apgar	Arterial pH umbilical cord
5-minute Apgar	$r = 0.607$, $p < 0.001$		
Arterial pH umbilical cord	$r = 0.267$, $p = 0.015$	$r = 0.333$, $p = 0.002$	
Venous pH umbilical cord	$r = 0.256$, $p = 0.018$	$r = 0.278$, $p = 0.010$	$r = 0.606$, $p < 0.001$

Supplementary Table 2: Comparisons between babies born at term (≥ 37 weeks of gestation) and preterm babies (gestational age < 37 weeks).

	**At term babies (n= 316)**	**Preterm babies (n=18)**	**p-value**
Birth weight (g)	3226.44 ± 528.55	2226.39 ± 691.39	<0.001
Weight percentile	48.38 ± 32.65	24.06 ± 32.71	0.002
1-minute Apgar	8.78 ± 0.70	8.50 ± 1.10	0.302
5- minute Apgar	9.67 ± 0.54	9.17 ± 0.62	<0.001

Supplementary Table 3: Maternal and neonatal thyroid function parameters according to intake of supplements, sex of new-born and prematurity.

	Maternal TSH (mIU/L)		Maternal FT4 (pmol/L)		Neonatal TSH (mIU/L)		Neonatal FT4 (pmol/L)	
Use of iodine supplements								
No	2.83 ± 1.60	*p* = 0.364	12.27 ± 3.12	*p* = 0.156	8.86 ± 7.31	*p* = 0.119	16.36 ± 2.97	*p* = 0.544
Yes	3.18 ± 2.26		13.26 ± 3.18		10.95 ± 7.60		16.66 ± 2.63	
Sex of the new-born								
Male	3.39 ± 2.40	*p* = 0.052	13.11 ± 2.78	*p* = 0.861	10.19 ± 9.78	*p* = 0.150	16.59 ± 3.06	*p* = 0.807
Female	2.87 ± 1.92		13.19 ± 3.54		9.66 ± 5.82		16.67 ± 2.20	
Gestational age								
Preterm	4.00 ± 2.57	*p* = 0.101	14.74 ± 5.91	*p* = 0.367	11.63 ± 5.61	*p* = 0.150	16.42 ± 3.09	*p* = 0.744
At term	3.08 ± 2.15		13.05 ± 2.98		10.87 ± 8.28		16.64 ± 2.63	
